# Isomorphic (Koebner) Phenomenon Induced by Insulin Analogue Injections in Psoriasis

**DOI:** 10.1210/jcemcr/luac016

**Published:** 2022-11-30

**Authors:** Saartje Thijs, Eric Balti, Corinne Degraeve, Peter Coremans

**Affiliations:** Department of Diabetes and Endocrinology, VITAZ Hospital, Sint-Niklaas, Belgium; Department of Diabetes and Endocrinology, VITAZ Hospital, Sint-Niklaas, Belgium; Molenstraat 160 Private Dermatology Clinic, Kruibeke, Belgium; Department of Diabetes and Endocrinology, VITAZ Hospital, Sint-Niklaas, Belgium

**Keywords:** diabetes mellitus, insulin therapy, injection sites, koebnerization, psoriasis

## Abstract

Koebner phenomenon is an uncommon skin-related complication of subcutaneous insulin injection in patients with diabetes mellitus. This reaction, also referred as isomorphic phenomenon, has previously been described in various conditions including vitiligo, lichen planus, and psoriasis. We report a 56-year-old woman insulin-treated patient with type 2 diabetes mellitus who developed new-onset, sharply well-demarcated erythematous scaly plaques at the insulin injection sites consistent with Koebner phenomenon. These lesions occurred after withdrawal of methotrexate initiated for the treatment of psoriasis. The lesions responded well to guselkumab, an interleukin-23 targeting agent but not ciclosporin. Of note, unlike previously reported cases, our patient developed isomorphic response under treatment with insulin analogues and during psoriasis flare-up. This case highlights the paramount role of thorough and cautious examination of injection and insertion sites in patients at risk undergoing subcutaneous continuous glucose monitoring or treated with continuous transdermal/subcutaneous insulin injections.

Lipohypertrophy is the most common skin-related complication of insulin therapy. Less common dermatological complications of insulin injection include lipoatrophy, localized allergic reaction, subcutaneous abscess, localized amyloidosis, and hyperpigmentation of the skin at the injection sites [1]. We describe the Koebner phenomenon in a patient with type 2 diabetes mellitus requiring insulin during a psoriasis flare-up. We discuss the case based on a literature search of previously described cases of koebnerization at insulin injection sites in patients with psoriasis.

## Case Report

A 56-year-old woman with type 2 diabetes for more than 30 years and treated with insulin for three years was evaluated at the diabetes clinic for routine follow-up. She also suffered from psoriasis for the last 22 years, for which she was managed with methotrexate 20 mg weekly, local calcipotriol 50 μg/g one application daily, and betamethasone dipropionate 0.05% twice daily. Eight months earlier, methotrexate was permanently withdrawn because of liver injury. Concerning her diabetes, lispro was used according to preprandial glucose values amounting to a total daily dose of 8 IU. A total of 14 IU of glargine was administered to cover the basal insulin requirements. Her other medications were atorvastatin 20 mg daily, aspirin 80 mg daily, and metformin 850 mg three times daily.

## Diagnostic Assessment

**Figure 1.**
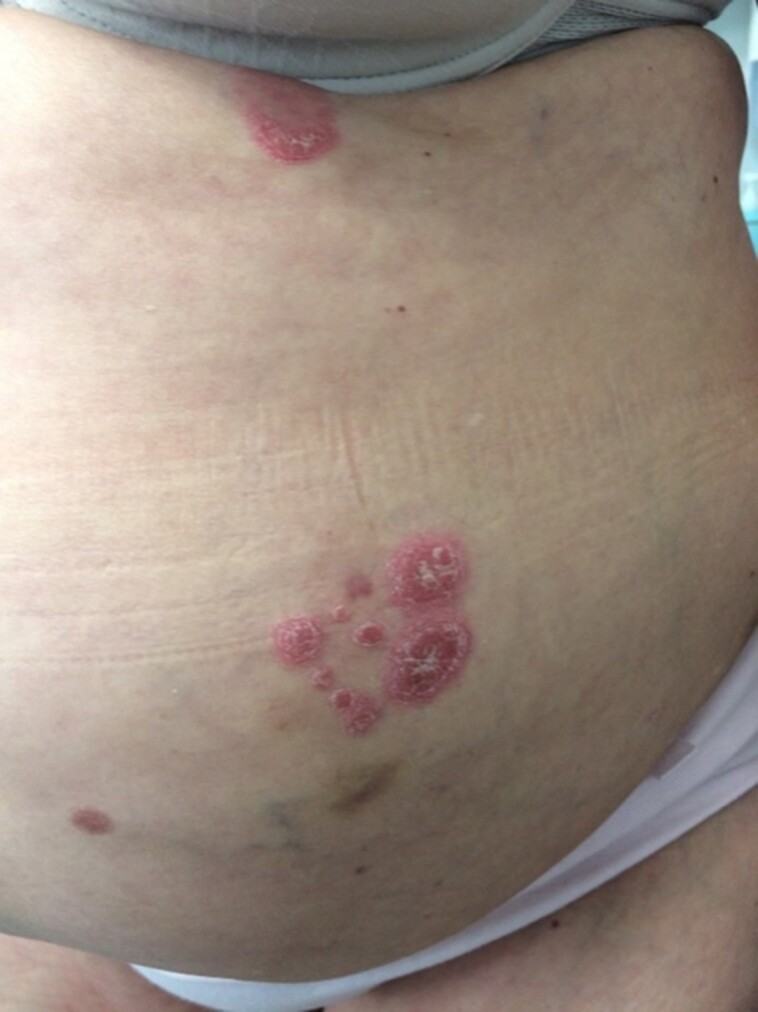
Psoriasis-associated Koebner phenomenon. After withdrawal of methotrexate, new-onset of sharply demarcated, erythematous, oval, red, and scaly plaques that differ in size at the insulin injection sites on the lower abdomen.

**Figure 2.**
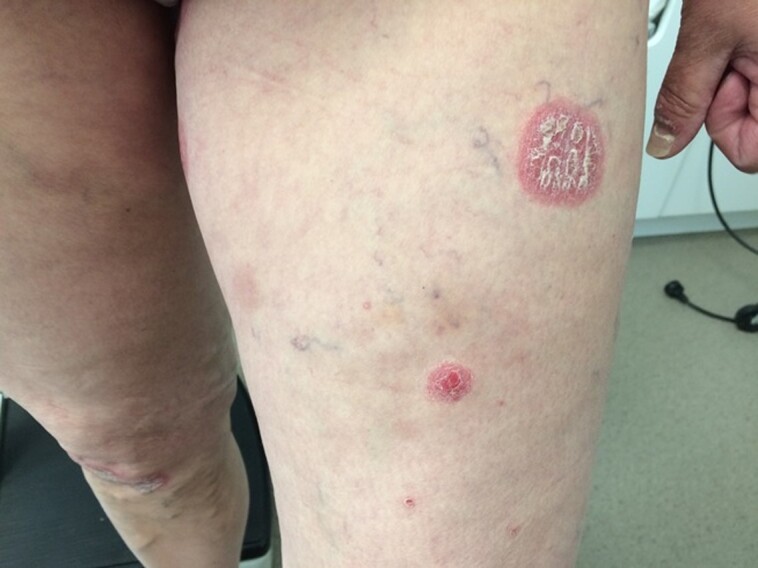
Psoriasis-associated Koebner phenomenon. After withdrawal of methotrexate, new-onset of sharply demarcated, erythematous, oval, red, and scaly plaques that differ in size at the insulin injection sites on both anterior proximal thighs.

We noticed a flare-up of the patient’s psoriasis including involvement of her nails, skin, localized at elbows, gluteal, lumbosacral, and submammary after withdrawal of methotrexate due to disturbed liver function tests. These plaques developed 48 to 72 hours after injection both with insulin lispro and glargine. In addition, she developed new sharply delineated, erythematous, oval, and scaly plaques of various sizes at the insulin injection sites on the lower abdomen (Fig. 1) and both anterior proximal thighs (Fig. 2).

Biological assessment revealed glycated hemoglobin A_1c_ of 7.0%. Both prandial and basal insulin requirements remained stable the last three months preceding the visit. Lipid profile, kidney, and liver function tests were within laboratory normal limits. Screening of microvascular complications revealed a stable, nonproliferative diabetic retinopathy.

We diagnosed psoriasis-associated Koebner phenomenon due to insulin injections.

## Treatment and Outcome

Besides calcipotriol and betamethasone dipropionate based on a consensual appreciation with colleagues of dermatology clinic, we initiated ciclosporin 100 mg twice daily. After two months, there was no substantial clinical improvement. We therefore started treatment with guselkumab, an interleukin-23 inhibitor. With remission starting after guselkumab administration, all plaques (including those at the injection sites) resolved completely and no new lesions developed six weeks after treatment initiation (after two injections).

## Discussion

The 2022 edition of the American Diabetes Association *Standards of Medical Care in Diabetes* recommends examination of insulin injection sites at every visit to rule out the presence of lipohypertrophy. Assessment of the correct use of injection devices and injection technique is a key component of a comprehensive medical evaluation and treatment plan in patients with diabetes mellitus [2]. A relatively recent meta-analysis revealed that the pooled prevalence of lipohypertrophy was 38% (95% CI, 29%-46%) among insulin-treated patients with diabetes mellitus. Less common dermatological complications of insulin injection include lipoatrophy, localized allergic reaction, subcutaneous abscess, localized amyloidosis, and hyperpigmentation at the site of injection [1].

Koebner phenomenon, also known as the isomorphic response, describes the appearance of new skin lesions of the same kind of preexisting dermatologic disease along sites of cutaneous injury. Although it could be observed in patients with vitiligo and lichen planus, Koebner phenomenon occurs more frequently in patients with psoriasis [3]. Overall, the prevalence of physician-diagnosed psoriasis has been estimated to be 1.92% in Western European countries [4]. Psoriasis can be associated with multiple comorbidities including diabetes mellitus. It has been suggested that chronic systemic inflammation occurring in the psoriatic state induces insulin resistance and therefore could increase the propensity to develop diabetes mellitus. In line with this, the relative risk for diabetes incidence in psoriasis has been estimated to be 1.50 (95% CI, 1.31-3.10) [5]. On the other hand, independently of comorbidities, the occurrence of new psoriatic lesions has been reported in approximately 25% to 30% of patients in an uninvolved skin region following injury [6]. This relates to the classical isomorphic response. Koebner phenomenon has already been described at insulin injection sites in diabetic patients with underlying dermatological diseases, such as vitiligo or lichen sclerosus atrophicus [7, 8]. To the best of our knowledge, there are only two other cases describing Koebner phenomenon in patients with psoriasis following insulin injections. Federman et al [9] described a 58-year-old diabetic male patient with developing multiple lichenified pink papules on the lower abdomen and proximal thighs at insulin injection sites. This guttate psoriatic flare cleared with topical application of clobetasol and calcipotriene and prevented the development of new lesions. Wang and Ran [10] reported an irregular, erythematous, scaling plaque with a well-defined boundary in the abdominal region after initiating therapy with human insulin injection in a 52-year-old woman. Histologic examination of these lesions revealed the typical features of psoriasis. In addition to the abdominal lesions, there were scaled erythematous plaques on both of her elbows and buttocks, without progressing and lasting for five years.

Our report is different from these two prior reports [9, 10] because those patients were treated with human insulin injection. We describe for the first time a patient with koebnerization due to injection with insulin analogues in the setting of known psoriasis. Whether the type of insulin used plays a role in the risk of isomorphic phenomenon warrants further investigation. Our patient developed the most common dermatological psoriasis phenotype at the injection sites, namely, plaques of psoriasis. Moreover, in previously reported cases, the authors described koebnerization during a latent disease stage, whereas in our patient this happened during a serious flare-up.

Our report “rings the bell” regarding the importance of a thorough inspection of all insulin injection sites, not only for excluding lipohypertrophy, but also to rule out other dermatological concerns such as isomorphic phenomenon. We provide additional evidence on the possibility of inducing new psoriasis lesions at insulin injection sites, the Koebner phenomenon. Thus, clinicians should be aware of this possibility in insulin-requiring diabetic patients with psoriasis. Therefore, we emphasize the importance of thoroughly inspecting all insulin injection sites and catheter insertion sites in insulin pump therapy, not only for excluding lipohypertrophy, but also for excluding other dermatological problems associated with insulin injections. We would not be surprised to see Koebner phenomenon emerging also in patients using subcutaneous continuous glucose monitoring systems or using the different delivery modalities of continuous transdermal/subcutaneous insulin.

## Learning Points

- Koebner phenomenon is an uncommon event in patients with psoriasis.
- In patients with diabetes, Koebner phenomenon could occur not only in patients with latent autoimmune disease including psoriasis, but also during exacerbations.
- Regular and cautious examination of insulin injection and device insertion sites is critical for the identification of new-onset lesions or an isomorphic phenomenon in patients with underlying dermatological diseases.

## Data Availability

Original data generated and analyzed during this study are included in this published article.
